# Indocyanine Green Loaded Reduced Graphene Oxide for In Vivo Photoacoustic/Fluorescence Dual-Modality Tumor Imaging

**DOI:** 10.1186/s11671-016-1288-x

**Published:** 2016-02-12

**Authors:** Jingqin Chen, Chengbo Liu, Guang Zeng, Yujia You, Huina Wang, Xiaojing Gong, Rongqin Zheng, Jeesu Kim, Chulhong Kim, Liang Song

**Affiliations:** Research Laboratory for Biomedical Optics and Molecular Imaging, Shenzhen Key Laboratory for Molecular Imaging, Institute of Biomedical and Health Engineering, Shenzhen Institutes of Advanced Technology, Chinese Academy of Sciences, Shenzhen, 518055 China; Beijing Center for Mathematics and Information Interdisciplinary Sciences (BCMIIS), Beijing, 100048 China; Department of Medical Ultrasound, The Third Affiliated Hospital of Sun Yat-sen University, Guangzhou, 510630 China; Departments of Creative IT Engineering and Electrical Engineering, Future IT Innovation Laboratory, Pohang University of Science and Technology (POSTECH), 77 Cheongam-Ro, Nam-Gu, Pohang, Gyeongbuk 790-784 Republic of Korea

**Keywords:** Reduced nano-graphene oxide, Indocyanine green, Photoacoustic/fluorescence dual-modality imaging, Molecular imaging

## Abstract

Multimodality imaging based on multifunctional nanocomposites holds great promise to fundamentally augment the capability of biomedical imaging. Specifically, photoacoustic and fluorescence dual-modality imaging is gaining much interest because of their non-invasiveness and the complementary nature of the two modalities in terms of imaging resolution, depth, sensitivity, and speed. Herein, using a green and facile method, we synthesize indocyanine green (ICG) loaded, polyethylene glycol (PEG)ylated, reduced nano-graphene oxide nanocomposite (rNGO-PEG/ICG) as a new type of fluorescence and photoacoustic dual-modality imaging contrast. The nanocomposite is shown to have minimal toxicity and excellent photoacoustic/fluorescence signals both in vitro and in vivo. Compared with free ICG, the nanocomposite is demonstrated to possess greater stability, longer blood circulation time, and superior passive tumor targeting capability. In vivo study shows that the circulation time of rNGO-PEG/ICG in the mouse body can sustain up to 6 h upon intravenous injection; while after 1 day, no obvious accumulation of rNGO-PEG/ICG is found in any major organs except the tumor regions. The demonstrated high fluorescence/photoacoustic dual contrasts, together with its low toxicity and excellent circulation life time, suggest that the synthesized rNGO-PEG/ICG can be a promising candidate for further translational studies on both the early diagnosis and image-guided therapy/surgery of cancer.

**Electronic supplementary material:**

The online version of this article (doi:10.1186/s11671-016-1288-x) contains supplementary material, which is available to authorized users.

## Background

Real-time near infrared (NIR) fluorescence imaging combined with passively or actively targeted NIR probes is becoming a promising approach that can be translated for clinical applications such as the identification of tumor margins intraoperatively [1–3]. To date, many groups have successfully demonstrated NIR fluorescence imaging with various NIR probes to identify neoplastic tissues in both preclinical and pilot clinical studies [4–7]. However, due to the strong optical scattering in biological tissue, the resolution of fluorescence imaging decreases dramatically as the imaging depth increases [8], which limits the effective imaging depth of this technology. Photoacoustic imaging is a rapidly growing hybrid imaging modality, which is based on the detection of acoustic waves upon the absorption of pulsed laser energy by either endogenous tissue chromophores or exogenous contrast agents [9, 10]. As the generated photoacoustic (ultrasonic) waves are generally two to three orders of magnitude less scattered in biological tissue compared with optical waves, photoacoustic imaging not only possesses the high sensitivity and specificity of optical imaging but also offers high spatial resolution at great depths (up to several centimeters) [11, 12]. While the spatial resolution at large depths of photoacoustic imaging is better than that of fluorescence imaging, the advantages of fluorescence imaging should not be diminished given its extremely high sensitivity and imaging speed for a large field-of-view. Hence, these two imaging modalities are complementary to each other in terms of imaging resolution, depth, sensitivity, and speed. As a result, for many molecular imaging applications, it is highly desirable to develop and synthesize photoacoustic/fluorescence dual-modality nanoprobes.

Because of its intrinsic absorption and emission maxima in the NIR region, indocyanine green (ICG) is regarded as an excellent dual-modality contrast agent for both fluorescence and photoacoustic imaging. Its NIR absorption maxima at 740 nm guarantees an effective penetration of light for both fluorescence and photoacoustic excitation, while the 800 nm fluorescence emission maxima permits a highly sensitive detection of the fluorescent photons generated in deep tissue. Moreover, ICG has been approved by the Food and Drug Administration (FDA) for a number of clinical applications [13, 14], such as ophthalmic imaging, and the assessment of cardiac output, hepatic functions, and blood flow [15]. However, ICG molecules are also known to have several major limitations for in vivo imaging, including concentration-dependent aggregation in aqueous solutions [16], and rapid clearance from the bloodstream [17]. To overcome these challenges, researchers have tried to incorporate ICG into poly(D, L–lactide–co–glycolide) (PLGA) and liposome to construct a nanoparticle delivery system [18] or combine ICG with graphene oxide (GO) to form a nanocomposite via *π*–*π* stacking interactions [19]. As a result, the stability and circulation time, as well as the biocompatibility of ICG, can be greatly improved. However, liposome is known to suffer from relatively poor stability, and the loading ratio of PLGA and GO delivery systems are considered to be relatively low [18, 20, 21].

During the past few years, reduced nano-graphene oxide (rNGO), a derivative of graphene oxide, has been used by many researchers to deliver aromatic molecules with high loading efficiency, due to its rich *π*–*π* conjugation structures [21–23]. Aromatic molecules such as doxorubicin (DOX), ICG, and camptothecin (CPT) can be effectively adsorbed on the rNGO surfaces by *π*–*π* stacking and hydrophobic interactions [24–26]. It is reported that functionalized rNGO can load more aromatic molecules than that of NGO and liposome [20, 21, 27, 28]. Moreover, rNGO has high optical absorption in the NIR regime. Thus, compared with other ICG delivery systems such as those based on PLGA or liposome, the photoacoustic signals at 780 nm (the absorption peak of ICG) can be greatly enhanced if rNGO is used as the carrier for ICG.

Conventionally, toxic chemical reducing reagents, such as sodium borohydride, hydrazine, and their derivatives, are used to reduce NGO. However, the rNGO produced by these methods often have agglomeration problems, and the residual toxic reduction reagents may also limit their in vivo applications [29–31]. Herein, we synthesized rNGO using a facile and green method as reported in our previous publication [32] and further loaded ICG onto the surfaces of rNGO with high efficiency to obtain a new type of multifunctional nanocomposite for both fluorescence and photoacoustic biomedical imaging applications. The ICG loaded rNGO-polyethylene glycol (PEG) (rNGO-PEG/ICG) nanocomposite showed excellent biocompatibility, high stability, long blood circulation time, and high NIR absorption. The loading efficiency of ICG (weight ratio of ICG to the rest of the nanocomposite) was also significantly higher compared with that of PLGA encapsulating ICG nanoparticles reported previously [18]. Therefore, for in vivo applications, the dose of the nanocomposite can be reduced to lower the potential risk of long-term toxicity. The synthesized rNGO-PEG/ICG was demonstrated as a new type of excellent multimodality contrast agent for both photoacoustic and fluorescence imaging in living mice. The results in the current study suggest that the rNGO-PEG/ICG fabricated using our green and facile method can be a promising candidate for further clinical translational studies on both the diagnosis and image-guided therapy/surgery of cancer in vivo.

## Methods

### Synthesis of rNGO-PEG/ICG

**Scheme 1:**
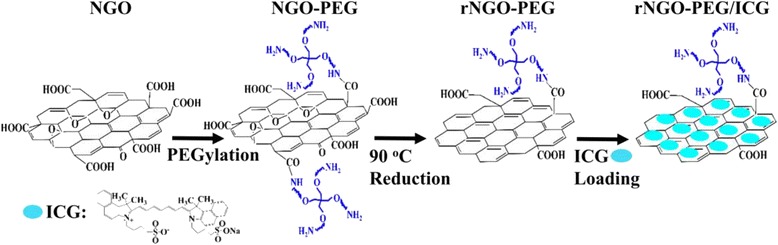
The synthesis route of rNGO-PEG/ICG from NGO. Step 1: PEGylation of NGO, step 2: reduction of NGO-PEG, step 3: loading ICG onto rNGO-PEG

Scheme 1 shows the synthesis strategy of rNGO-PEG/ICG. The NGO-PEG was synthesized following the method as reported by us previously [32]. In brief, GO powder was made using a modified Hummer’s method with expandable graphite flakes (XF NANO Co., Ltd. China). NGO solution was prepared by sonicating the GO powder. NaOH (1.2 g) and Cl–CH_2_–COOH (1.0 g) were added to the NGO suspension (~2 mg mL^−1^) and sonicated for 30 min to obtain carboxylation NGO (NGO–COOH). The resulting NGO–COOH suspension was neutralized and purified by multiple rounds of rinsing and filtration. Four-arm polyethylene glycol-Amine (5 kDa MW, Creative PEG Works, USA) and NGO-COOH suspension were reacted at a pH of 6, and then, 1-ethyl-3-(3-dimethylaminopropyl) carbodiimide (4 mmol L^−1^) and *N*-hydroxysuccinimide (10 mmol L^−1^) (EDC and NHS, Sigma) were added to the above suspension. The produced NGO-PEG was purified by centrifuging with 30 kDa ultracentrifuge tubes (Millipore) and dialyzed against distilled water for overnight to remove any ions. Then, the solution was centrifuged at 12,000*g* for 30 min to remove any unstable aggregates. After that, 50 mL of the NGO-PEG solution (1 mg mL^−1^) was transferred to a sealed glass bottle and then bathed at 90 °C for 24 h. Finally, the resulting rNGO-PEG solution was centrifuged at 6000*g* for 10 min to remove any unstable aggregates and stored at 4 °C for further use.

Loading of ICG onto rNGO-PEG was implemented by first adding sufficient ICG (~20 mg) to rNGO-PEG aqueous suspension (5 mL, 1 mg mL^−1^) and then stirring the mixture at 4 °C overnight. The product was repeatedly filtered through a 30 kDa filter (Millipore) and centrifuged at 6000*g* to remove unbound ICG molecules. The ultimately produced rNGO-PEG/ICG solution was stored at 4 °C for further use. As a control, ICG was also loaded onto unreduced NGO-PEG at the same condition to produce NGO-PEG/ICG. To estimate the amount of ICG loaded onto NGO-PEG and rNGO-PEG, we first measured the absorbance of NGO-PEG/ICG and rNGO-PEG/ICG solutions at 780 nm and then subtracted the counterparts of NGO-PEG and rNGO-PEG, respectively. After that, we compared the obtained absorbance with the standard curve of free ICG to estimate the concentration of the loaded ICG. The standard curve here means the correlation between the ICG concentration and its absorbance at the absorption peak, given that the ICG concentration is in the linear range.

### Characterizations

Dynamic light scattering (DLS) analysis was performed using a Zetasizer Nano ZS system (Malvern Instruments) to characterize the average hydrodynamic diameter. The morphology of the materials was imaged with atomic force microscopy (AFM, Agilent Technologies 5500, USA) and transmission electron microscopy (TEM, FEI Tecnai G2 F20 S-Twin, USA). To analyze the loading ratio of ICG, UV–vis spectra were acquired by an UV–vis spectrometer (Lambda 25, PerkinElmer, USA), with the sample in a 1-cm quartz cuvette. Luminescence spectrometer (F900 Edinburgh Instruments Ltd, Livingston, UK) was used to measure fluorescence emission of the sample. To confirm the success of covalent PEG conjugation to NGO as well as the reduction of NGO-PEG to rNGO-PEG, Fourier transfer infrared (FT-IR) spectra were recorded on a Magna-IR 750 FT-IR spectrometer (Nicolet, USA). X-ray photoelectron spectroscopy (XPS) measurement was carried out with an ESCALAB 250XI high performance electron spectrometer (Thermo, USA). All photoacoustic measurements were conducted with our custom-built photoacoustic imaging system, which will be described in the “Photoacoustic and fluorescence imaging systems” section.

### Cell Culture and Cellular Uptake of rNGO-PEG/ICG

A Hela cell line (Helen Lake) was obtained from American Type Culture Collection and cultured at 37 °C in Dulbecco’s modified Eagle’s medium (DMEM, Gibco, Grand Island, NY, USA) supplemented with 10 % fetal calf serum (FCS, Gibco) and 5 % CO_2_ in a humidified incubator.

For the cellular uptake study of rNGO-PEG/ICG, Hela cells were cultured in the confocal dish for 24 h and then incubated with free ICG and rNGO-PEG/ICG suspension (with 30 μg mL^−1^ ICG) for 3 h. After that, the cells were rinsed with phosphate-buffered saline (PBS) for three times to remove the redundant nanocomposites and subsequently fixed with 4 % paraformaldehyde solution for 10 min. Then, the cells were washed with PBS again and stained by 4′,6-diamidino-2-phenylindole (DAPI, 10 μg mL^−1^) for 2–3 min. Finally, the stained cells were imaged with a confocal laser scanning microscope (CLSM, Leica TCS SP5, Wetzlar, Germany), using 405 and 633 nm laser wavelength, respectively, for DAPI and ICG excitation. In addition, the cell uptake ratios of free ICG and rNGO-PEG/ICG were analyzed using the flow cytometry. In brief, the cells were incubated with rNGO-PEG/ICG for 3 h and then washed by PBS three times. After digesting the cells, the ICG fluorescence signals in cells were detected and analyzed by flow cytometry.

### Animal and Tumor Model

Female BALB/c nude mice (4–6 weeks old) were obtained from the Medical Experimental Animal Center of Guangdong Province (Guangzhou, China). All animal experiment procedures were approved by the Animal Study Committee of the Shenzhen Institutes of Advanced Technology, Chinese Academy of Sciences. The Hela tumor models were established by subcutaneously injecting 5 × 10^6^ cells in PBS (200 μL) into the back of BALB/c nude mice. When the tumor size reached approximately 100 mm^3^, the mice were divided into different groups (five mice per group) for subsequent experiments.

### Photoacoustic and Fluorescence Imaging Systems

An acoustic-resolution photoacoustic microscopy (AR-PAM) system was used for all the photoacoustic measurements in this study. The details of the system are reported in our earlier publication [18]. In brief, the AR-PAM system consists of a tunable pulsed optical parametric oscillator (OPO) laser (Vibrant 355 II HE, Opotek, Carlsbad, USA), a focused ultrasound transducer (V315-SU, Olympus IMS, Waltham, USA; central frequency: 10 MHz; fractional bandwidth: 6 MHz; N.A.: 0.4), and a precision motorized 3D scanning stage (PSA2000-11, Zolix, Beijing, China). During experiments, the OPO laser was operated at 780 nm (peak absorption wavelength of ICG), with a repetition rate of 10 Hz and a pulse width of 5 ns. The laser fluence is 2 mJ cm^−2^ for phantom study and 6 mJ cm^−2^ for in vivo imaging experiments.

For fluorescence imaging, a commercial three dimensional imaging system (CRi MaestroTM, USA) was used to acquire the images of both phantom and animal studies.

### In Vitro Toxicity Study

For cytotoxicity assay, approximately 5 × 10^4^ Hela cells/well were plated in a 96-well plate with medium (100 μL) in each well and cultured for 24 h. After that, various concentrations of rNGO-PEG/ICG were added to the wells. The relative cell viability was assessed by Cell Counting Kit-8 (CCK-8, Dojindo, Japan) assay after incubating for 24 and 48 h, respectively.

### Histology Analysis

rNGO-PEG/ICG (200 μL, ~15 mg kg^−1^ concentration) was intravenously injected into five healthy female BALB/c nude mice. Another five healthy BALB/c nude mice were used as the untreated control, in which the same amount of saline was injected intravenously. Then, all the mice were sacrificed; the major organs of these mice were harvested, fixed in 10 % neutral buffered formalin, processed into paraffin, sectioned at a thickness of 5–10 μm, stained with hematoxylin & eosin (H&E), and examined under a digital optical microscope (Cx41, Olympus).

## Results and Discussion

### Synthesis and Characterization of rNGO-PEG

**Fig. 1:**
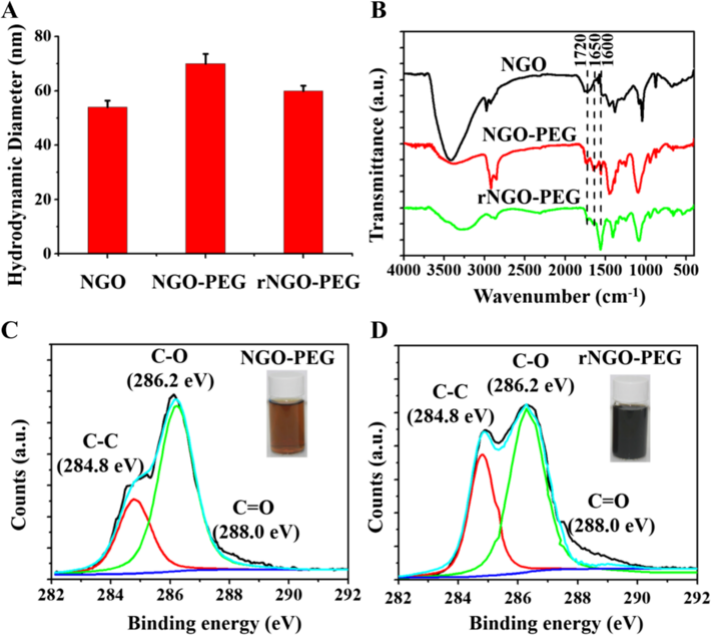
Reduction of NGO-PEG. **a** The average hydrodynamic diameter of NGO, NGO-PEG, and rNGO-PEG. **b** FT-IR spectra of NGO, NGO-PEG, and rNGO-PEG. **c**, **d** XPS spectra of NGO-PEG and rNGO-PEG. *Inset*: Photographs of NGO-PEG and rNGO-PEG solutions

As shown in Fig. 1a, the average hydrodynamic diameters of NGO, NGO-PEG, and rNGO-PEG are about 55, 72, and 63 nm, respectively, measured with dynamic light scattering. FT-IR spectra confirmed the success of covalent PEG conjugation to NGO as well as the reduction of NGO-PEG to rNGO-PEG (Fig. 1b). The additional peaks at ~1650 cm^−1^ in both the NGO-PEG and rNGO-PEG spectra compared with that of NGO indicated the existence of −NH_2_ groups, suggesting that the PEG functional groups were conjugated to NGO successfully and the reduction process did not cause a significant loss of PEG from rNGO-PEG. As a result, the biocompatibility of both NGO-PEG and rNGO-PEG was significantly improved compared with that of NGO. The increase at the peak of ~1625 cm^−1^ (C=C) of rNGO-PEG compared with that of NGO-PEG indicated the restoration of *π−π* structure [33], and the decrease at the peak of ~1720 cm^−1^ indicated the removal of C=O (i.e., oxygen groups) [34]. Both changes confirmed that the NGO-PEG was successfully reduced [33, 34].

XPS spectra measurements were also carried out to provide further proof of the reduction process from NGO-PEG to rNGO-PEG. The diminished C–O signal (286.8 eV) and increased C=O (288.0 eV) and C–C signals (284.6 eV) (Fig. 1c, d) demonstrated the successful reduction of NGO [35, 36], likely due to the strong reducibility of the four-armed PEG-NH_4_ originated from its −NH_2_ groups [37, 38]. The insets of Fig. 1c, d showed the color change of NGO-PEG solution from brown to dark black, which was an additional intuitive evidence for the reduction of NGO [39].

### Characterization of rNGO-PEG/ICG

**Fig. 2:**
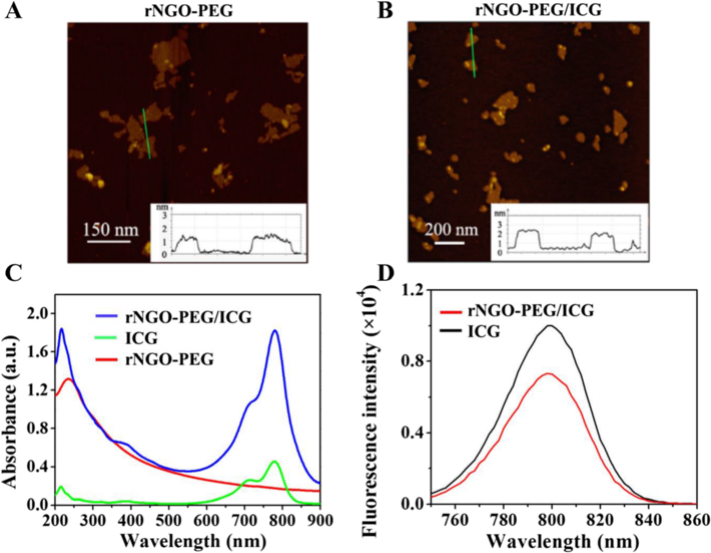
Synthesis and characterization of rNGO-PEG/ICG. **a**, **b** AFM images of rNGO-PEG and rNGO-PEG/ICG. **c** UV–vis absorption spectra of free ICG, rNGO-PEG, and rNGO-PEG/ICG solutions. **d** Fluorescence spectra of ICG and rNGO-PEG/ICG solutions with the same concentration of ICG

The morphology of the fabricated rNGO-PEG/ICG was imaged by AFM. As shown in Fig. 2b and Additional file 1: Figure S1A, rNGO-PEG/ICG exhibited a lamellar structure with a thickness of about 2.2 nm, which was larger than that of rNGO-PEG (about 1.6 nm, Fig. 2a), likely owing to the ICG physical adsorption onto rNGO-PEG. In addition, after exposing under the 780 nm laser (6 mJ cm^−2^) for 30 min (Additional file 1: Figure S1), the morphology of rNGO-PEG/ICG had no significant change, demonstrating the great photostability of rNGO-PEG/ICG. UV–vis spectra showed that the absorbance of rNGO-PEG had increased by ~threefold over that of NGO-PEG in the NIR range (700–900 nm) (Fig. 2c and Additional file 1: Figure S2A). In addition, the feature peak (at ~780 nm) of ICG absorption can be seen in the spectra of rNGO-PEG/ICG (Fig. 2c), indicating that ICG was successfully loaded onto rNGO-PEG via *π*–*π* stacking and hydrophobic interactions. The loading capacity of rNGO-PEG (about 180 % *w*/*w*) was about twofold than that of NGO/PEG (about 86 % *w*/*w*) (Additional file 1: Figure S2B), which was probably because of the repaired aromatic ring structures that increased the *π*–*π* stacking and hydrophobic interactions between rNGO-PEG and the aromatic ICG molecules. Figure 2d shows that the fluorescence spectrum of rNGO-PEG/ICG agrees well with that of free ICG of the same ICG concentration, and the fluorescence intensity decrease of rNGO-PEG/ICG compared with that of free ICG is presumably due to the photo quenching of ICG upon conjugation to rNGO. This result indicates that the loading process did not cause significant perturbation to the fluorescence property of ICG. To evaluate the stability of rNGO-PEG/ICG, we monitored the dispersions of rNGO-PEG/ICG in water, cell medium (DMEM), fetal bovine serum (FBS), and PBS solutions. The results showed that no obvious aggregation was found in the solutions and rNGO-PEG/ICG remained stable for about 1 month at 4 °C (Additional file 1: Figure S3).

### In Vitro Cellular Uptake

**Fig. 3:**
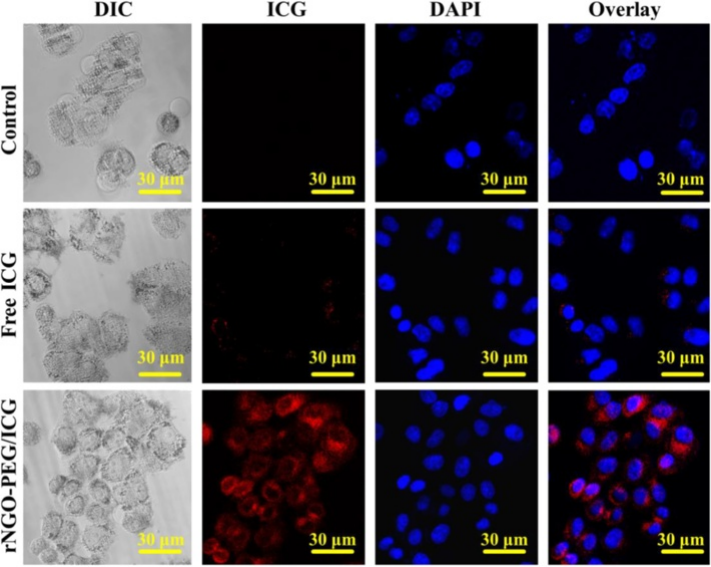
Confocal fluorescence microscopic images of Hela cells treated with PBS (as blank control), free ICG, and rNGO-PEG/ICG for 3 h. The cell nuclei were stained by DAPI

To confirm the uptake of rNGO-PEG/ICG by tumor cells, Hela cells incubated with free ICG and rNGO-PEG/ICG were observed under a commercial confocal laser scanning microscope. As shown in Fig. 3, compared with the blank control and free ICG treated groups, the rNGO-PEG/ICG treated group has shown a significant fluorescence of ICC surrounding the DAPI stained Hela nuclei. Additional file 1: Figure S4 shows that the cells cultured with free ICG and rNGO-PEG/ICG, respectively, had 9.1 and 63.5 % of uptake ratio. The high efficient cell uptake of rNGO-PEG/ICG may be beneficial for cancer therapy.

### Photoacoustic Imaging of Phantoms

**Fig. 4:**
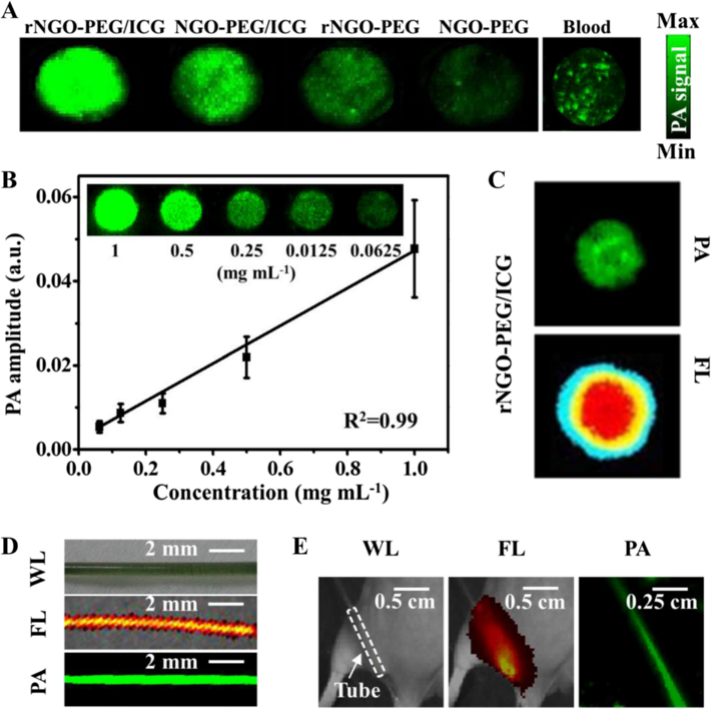
Photoacoustic (*PA*) imaging of various phantom samples, as well as comparisons between photoacoustic and fluorescence (*FL*) imaging. **a** Photoacoustic images of NGO-PEG, rNGO-PEG, NGO-PEG/ICG, and rNGO-PEG/ICG with the same GO concentration and whole blood. **b** The linear relationship between PA signal intensity and the concentration of rNGO-PEG/ICG. *Inset*: Photoacoustic images of various concentrations of rNGO-PEG/ICG. **c** Photoacoustic and FL images of rNGO-PEG/ICG covered with 5-mm-thick agarose gel containing 0.5 % intralipid. **d** White light (*WL*), FL, and PA MAP images of a PE tube filled with rNGO-PEG/ICG. **e** White light, FL, and PA MAP images of a mouse with the rNGO-PEG/ICG filled PE tube implanted subcutaneously at the dorsal aspect of the leg. The *white dash box* indicates the location of the tube

Figure 4 shows the AR-PAM results of various phantoms with the excitation wavelength tuned at 780 nm. Figure 4a shows the maximum amplitude projection (MAP) photoacoustic images of NGO-PEG, rNGO-PEG, NGO-PEG/ICG, and rNGO-PEG/ICG phantoms with the same NGO concentration and whole blood as a control. It can be seen that (1) the photoacoustic signal intensity of rNGO-PEG was stronger than that of NGO-PEG because of the optical absorption enhancement in the NIR range after reduction; (2) compared with that of NGO-PEG and rNGO-PEG, the photoacoustic signal intensity of both NGO-PEG/ICG and rNGO-PEG/ICG were significantly enhanced by the distinct absorption of ICG at 780 nm; and (3) compared with NGO-PEG/ICG and blood, the photoacoustic signal of rNGO-PEG/ICG was much stronger, primarily because of the higher ICG loading efficiency onto rNGO-PEG (also because of the enhanced absorption upon reduction from NGO-PEG to rNGO-PEG). Figure 4b shows that the photoacoustic signal is increasing linearly (*R*^2^ = 0.99) with the rNGO-PEG/ICG concentration, within the range (≤1 mg mL^−1^) used in our measurements. In addition, the MAP photoacoustic images of the phantoms are also shown in the inset of Fig. 4b.

### Fluorescence and Photoacoustic Imaging of Phantoms

Figure 4c shows both the photoacoustic and fluorescence images of a rNGO-PEG/ICG phantom covered with a 5-mm-thick agarose gel containing 0.5 % intralipid to mimic the optical scattering of biological tissue. It can be seen that the photoacoustic image presents a much sharper boundary of the target in this case. Further, fluorescence and photoacoustic MAP images of a polyethylene (PE) tube filled with rNGO-PEG/ICG were acquired under two circumstances: (1) in non-scattering medium (water) and (2) when the tube was implanted subcutaneously into a mouse leg. As shown in Fig. 4d, when there is no strong optical scattering, both the fluorescence and photoacoustic MAP images agree well with the photograph of the tube (i.e., the real shape of the tube). However, when the PE tube was subcutaneously implanted into the leg of a living mouse and thus covered by its skin, muscle, and connective tissue, the fluorescence image was seriously blurred due to the strong optical scattering. On the contrast, the photoacoustic image was still able to clearly delineate the shape of the tube (Fig. 4e), demonstrating the unique advantage of photoacoustic technology for high resolution imaging at a relatively large depth in scattering biological tissue.

### In Vivo Fluorescence and Photoacoustic Imaging

**Fig. 5:**
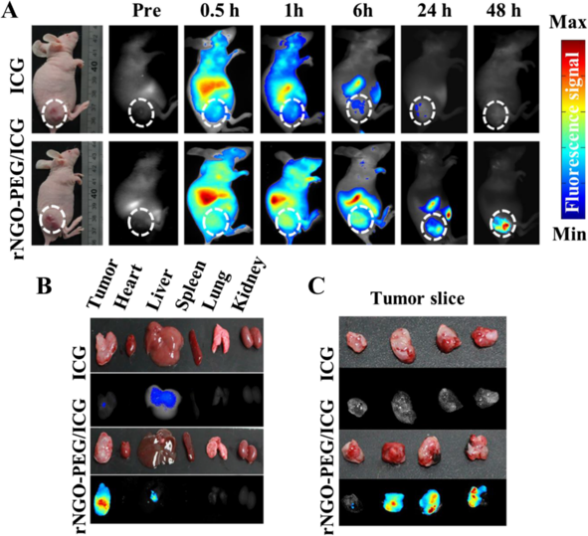
In vivo fluorescence imaging. **a** Fluorescence signal distribution within the tumor-bearing mouse body as a function of time after tail vein injection of ICG and rNGO-PEG/ICG. *White dash line* encircles the tumor region. **b**, **c** The *ex vivo* fluorescence signal of the excised major organs (tumor, heart, liver, spleen, lung, and kidney) and tumor slices of mice at 48 h post injection

Fluorescence imaging was carried out to investigate the biodistribution of rNGO-PEG/ICG in tumor-bearing mice. Figure 5 depicts a typical fluorescence signal distribution within the mouse body as a function of time after tail vein injection of rNGO-PEG/ICG (150 μL, ~15 mg kg^−1^) and free ICG (as the control group, 10 mg kg^−1^). For both rNGO-PEG/ICG and free ICG, it was found that (Fig. 5a) the signals started to gradually accumulate towards internal organs such as the liver and intestine from 0.5 to 6 h (during this period, a significant accumulation in the tumor region was also found in the rNGO-PEG/ICG group). More importantly, along with time, for the rNGO-PEG/ICG group, the accumulation of the material towards the tumor region was becoming more and more significant—after 48 h; well detectable fluorescence signals were found only in the tumor region. For the free ICG control group, as expected, the clearance process was rather rapid, and the majority of the dye was cleared out of the body after ~6 h, with no significant accumulation observed in the tumor region. These results suggest that, compared with free ICG, rNGO-PEG/ICG has greatly enhanced the permeability and retention (EPR) effect to enable effective imaging of tumor in vivo. To further validate these results, we excised the tumors and harvested the major organs of the mice at 48 h post injection of the samples. Figure 5b shows the fluorescence signals of the excised major organs (heart, liver, spleen, lung, and kidney) and tumors from the tumor-bearing mice 48 h after tail vein injection of the rNGO-PEG/ICG and free ICG; Fig. 5c further shows the fluorescence signals of the tumor slices. From both Fig. 5b, c, it can be seen that significant fluorescence signals can only be observed in the tumors of the rNGO-PEG/ICG-injected mice, but not the free ICG-injected mice.

**Fig. 6:**
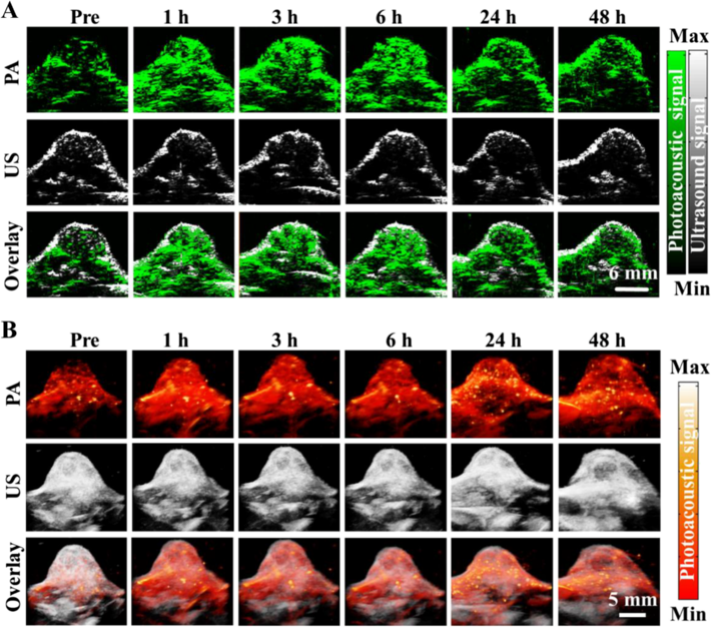
In vivo photoacoustic imaging. **a** B-scan and **b** 3D ultrasound (*US*) and PA images of the tumor region obtained at 1, 3, 6, 24, and 48 h post tail vein injection of rNGO-PEG/ICG. The US images delineated the skin and tumor boundaries, while the PA images showed the accumulation and distribution of the nanocomposite within the tumor region

Figure 6a, b, respectively, shows the cross-sectional and three-dimensional ultrasonic, photoacoustic, and fused images of the tumor region as a function of time, upon the tail vein injection of rNGO-PEG/ICG (150 μL, ~15 mg kg^−1^). In addition to the position and rough contour of the tumor that can be identified with ultrasound imaging, photoacoustic images further show the depth-resolved rNGO-PEG/ICG distribution in the tumor region at high resolution. Such information can be vital for both image-guided tumor therapy and for assessing certain functional characteristics (e.g., vascularization) of cancer. As can be seen, the dynamics of the photoacoustic signal in the tumor region exhibits similar features to that of fluorescence imaging (Fig. 5a).

It was reported that, in order to take full advantage of the EPR effect, the hydrodynamic diameter (HD) of the nanoparticles should be in the range of 10 to 200 nm, and the nanoparticles should be of a hydrophilic (polar or zwitterionic) surface coating for an extended blood circulation time and undesirable clearance by the immune system [40–42]. In our experiments, excellent passive tumor-targeting capability of rNGO-PEG/ICG nanocomposite has been demonstrated through both fluorescence and photoacoustic imaging, which may be attributed to the following two reasons: (1) the 80-nm diameter is within the optimal hydrodynamic size range for efficient delivery and accumulation of the nanocomposite in the tumor region and (2) the PEG layer on the surface of rNGO-PEG/ICG can effectively reduce its clearance by the reticuloendothelial system, resulting in a prolonged blood circulation time.

### In Vitro and In Vivo Toxicity Assessment

**Fig. 7:**
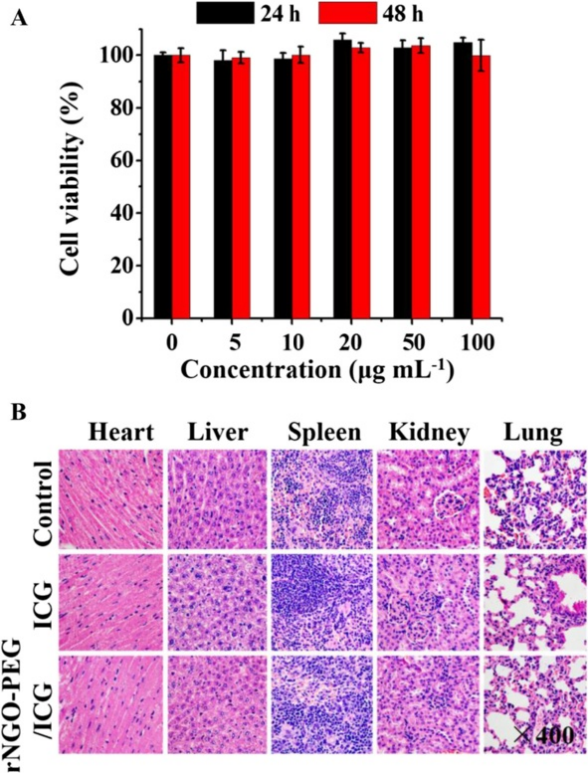
In vitro and in vivo toxicity study of rNGO-PEG/ICG. **a** Cytotoxicity of rNGO-PEG/ICG (with 0–100 μg mL^−1^) against Hela cells by CCK-8 method. Cells were incubated with rNGO-PEG/ICG for 24 and 48 h, respectively. **b** Representative H&E-stained images of major organs including the heart, liver, spleen, kidney, and lung collected from mice sacrificed 7 days after injection of rNGO-PEG/ICG

The toxicity of rNGO-PEG/ICG was assessed through both in vitro and in vivo experiments. As shown in Fig. 7a, no significant decrease of cell viability is observed at 24 and 48 h after the treatment of rNGO-PEG/ICG with a concentration varying from 0 to 100 μg mL^−1^. Figure 7b shows the H&E staining of the major organs (heart, liver, spleen, kidney, and lung) collected from a representative mouse on day 7 after the injection of rNGO-PEG/ICG and free ICG. Compared with the free ICG and blank control groups, no obvious tissue damages are shown in the rNGO-PEG/ICG group.

## Conclusions

Utilizing a green reduction strategy, we fabricated rNGO-PEG with excellent biocompatibility. Further, rNGO-PEG/ICG nanocomposite was synthesized by efficiently loading ICG onto the rNGO-PEG surfaces via *π*–*π* stacking. The developed nanocomposite was shown to possess high stability, strong fluorescence, and greatly enhanced NIR absorption over either ICG or rNGO-PEG alone. As a result, it can be an excellent agent for photoacoustic/fluorescence dual-modality imaging. In vitro studies showed that the nanocomposite could be efficiently uptaken by tumor cells without causing a significant decrease of cell viability. Moreover, upon tail vein injection of rNGO-PEG/ICG into tumor-bearing mice, significant contrast enhancement was seen in the tumor regions in both photoacoustic and fluorescence images acquired in vivo, throughout the entire imaging period. Overall, our results show that the developed rNGO-PEG/ICG nanocomposite is capable of effectively targeting tumor in vivo via the EPR effect. Meanwhile, it provides high contrasts for both fluorescence and photoacoustic imaging. The excellent passive targeting and imaging properties, together with its low toxic demonstrated in vitro and in vivo, suggest that the rNGO-PEG/ICG nanocomposite synthesized in this work can be of great potential for further clinical translation for both the diagnosis and image-guided therapy of cancer.

## Additional file

Additional file 1:
**The supplementary characterization results of rNGO-PEG/ICG.** (DOCX 2405 kb)

## Abbreviations

CCK-8: Cell Counting Kit-8
FL: fluorescence
GO: graphene oxide
ICG: indocyanine green
PA: photoacoustic
PEG: polyethylene glycol

## References

[1] Shinoda J, Yano H, Yoshimura S, Okumura A, Kaku Y, Iwama T, Sakai N (2003). Fluorescence-guided resection of glioblastoma multiforme by using high-dose fluorescein sodium: technical note. J Neurosurg.

[2] Gao X, Cui Y, Levenson RM, Chung LW, Nie S (2004). In vivo cancer targeting and imaging with semiconductor quantum dots. Nat Biotechnol.

[3] Liu J, Yu M, Zhou C, Yang S, Ning X, Zheng J (2013). Passive tumor targeting of renal-clearable luminescent gold nanoparticles: long tumor retention and fast normal tissue clearance. J Am Chem Soc.

[4] Van Dam GM, Themelis G, Crane LM, Harlaar NJ, Pleijhuis RG, Kelder W, Sarantopoulos A, de Jong JS, Arts HJ, van der Zee AG, Bart J, Low PS, Ntziachristos V (2011). Intraoperative tumor-specific fluorescence imaging in ovarian cancer by folate receptor-α targeting: first in-human results. Nat Med.

[5] Troyan SL, Kianzad V, Gibbs-Strauss SL, Gioux S, Matsui A, Oketokoun R, Ngo L, Khamene A, Azar F, Frangioni JV (2009). The FLARE™ intraoperative near-infrared fluorescence imaging system: a first-in-human clinical trial in breast cancer sentinel lymph node mapping. Ann Surg Oncol.

[6] Nguyen QT, Tsien RY (2013). Fluorescence-guided surgery with live molecular navigation—a new cutting edge. Nat Rev Cancer.

[7] Li K, Liu B (2014). Polymer-encapsulated organic nanoparticles for fluorescence and photoacoustic imaging. Chem Soc Rev.

[8] Xi L, Zhou G, Gao N, Yang L, Gonzalo DA, Hughes SJ, Jiang H (2014). Photoacoustic and fluorescence image-guided surgery using a multifunctional targeted nanoprobe. Ann Surg Oncol.

[9] Bai X, Gong X, Hau W, Lin R, Zheng J, Liu C, Zeng C, Zou X, Zheng H, Song L (2014). Intravascular optical-resolution photoacoustic tomography with a 1.1 mm diameter catheter. PLoS One.

[10] Song KH, Wang LV (2007). Deep reflection-mode photoacoustic imaging of biological tissue. J Biomed Opt.

[11] Weissleder R (2006). Molecular imaging in cancer. Science.

[12] Wang LV, Hu S (2012). Photoacoustic tomography: in vivo imaging from organelles to organs. Science.

[13] Benson RC, Kues HA (1978). Fluorescence properties of indocyanine green as related to angiography. Phys Med Biol.

[14] Desmettre T, Devoisselle JM, Mordon S (2000). Fluorescence properties and metabolic features of indocyanine green (ICG) as related to angiography. Surv Ophthalmol.

[15] Xu G, Piao D, Musgrove CH, Bunting CF, Dehghani H (2008). Trans-rectal ultrasound-coupled near-infrared optical tomography of the prostate, part I: simulation. Opt Express.

[16] Manchanda R, Fernandez-Fernandez A, Nagesetti A, McGoron AJ (2010). Preparation and characterization of a polymeric (PLGA) nanoparticulate drug delivery system with simultaneous incorporation of chemotherapeutic and thermo-optical agents. Colloid Surface B.

[17] Rudin M (2009). Noninvasive structural, functional, and molecular imaging in drug development. Curr Opin Chem Biol.

[18] Wang H, Liu C, Gong X, Hu D, Lin R, Sheng Z, Zheng C, Yan M, Chen J, Cai L, Song L (2014). In vivo photoacoustic molecular imaging of breast carcinoma with folate receptor-targeted indocyanine green nanoprobes. Nanoscale.

[19] Wang YW, Fu YY, Peng QL, Guo SS, Liu G, Li J, Yang HH, Chen GN (2013). Dye-enhanced graphene oxide for photothermal therapy and photoacoustic imaging. J Mater Chem B.

[20] Chen YW, Chen PJ, Hu SH, Chen IW, Chen SY (2014). NIR-triggered synergic photo-chemothermal therapy delivered by reduced graphene oxide/carbon/mesoporous silica nanocookies. Adv Funct Mater.

[21] Kim H, Lee D, Kim J, Kim TI, Kim WJ (2013). Photothermally triggered cytosolic drug delivery via endosome disruption using a functionalized reduced graphene oxide. ACS Nano.

[22] Su Y, Du J, Sun D, Liu C, Cheng H (2013). Reduced graphene oxide with a highly restored π-conjugated structure for inkjet printing and its use in all-carbon transistors. Nano Res.

[23] Chen J, Liu H, Zhao C, Qin G, Xi G, Li T, Wang X, Chen T (2014). One-step reduction and PEGylation of graphene oxide for photothermally controlled drug delivery. Biomaterials.

[24] Gonçalves G, Vila M, Portolés MT, Vallet-Regi M, Gracio J, Marques PA (2013). Nano-graphene oxide: a potential multifunctional platform for cancer therapy. Adv Healthc Mater.

[25] Miao W, Shim G, Kim G, Lee S, Lee HJ, Kim YB, Byun Y, Oh YK (2015). Image-guided synergistic photothermal therapy using photoresponsive imaging agent-loaded graphene-based nanosheets. J Control Release.

[26] Liu Z, Robinson JT, Sun X, Dai H (2008). PEGylated nanographene oxide for delivery of water-insoluble cancer drugs. J Am Chem Soc.

[27] Zheng XT, Li CM (2012). Restoring basal planes of graphene oxides for highly efficient loading and delivery of β-lapachone. Mol Pharmaceut.

[28] Wang Y, Wang K, Zhao J, Liu X, Bu J, Yan X, Huang R (2013). Multifunctional mesoporous silica-coated graphene nanosheet used for chemo-photothermal synergistic targeted therapy of glioma. J Am Chem Soc.

[29] Robinson JT, Tabakman SM, Liang Y, Wang H, Casalongue HS, Vinh D, Dai H (2011). Ultrasmall reduced graphene oxide with high near-infrared absorbance for photothermal therapy. J Am Chem Soc.

[30] Yang K, Wan J, Zhang S, Tian B, Zhang Y, Liu Z (2012). The influence of surface chemistry and size of nanoscale graphene oxide on photothermal therapy of cancer using ultra-low laser power. Biomaterials.

[31] Shin HJ, Kim KK, Benayad A, Yoon SM, Park HK, Jung IS, Jin MH, Jeong HK, Kim JM, Choi JY, Lee YH (2009). Efficient reduction of graphite oxide by sodium borohydride and its effect on electrical conductance. Adv Funct Mater.

[32] Chen J, Wang X, Chen T (2014). Facile and green reduction of covalently PEGylated nanographene oxide via a “water-only” route for high-efficiency photothermal therapy. Nanoscale Res Lett.

[33] Guo Y, Sun X, Liu Y, Wang W, Qiu H, Gao J (2012). One pot preparation of reduced graphene oxide (RGO) or Au (Ag) nanoparticle-RGO hybrids using chitosan as a reducing and stabilizing agent and their use in methanol electrooxidation. Carbon.

[34] Kuila T, Bose S, Mishra AK, Khanra P, Kim NH, Lee JH (2012). Chemical functionalization of graphene and its applications. Prog Mater Sci.

[35] Akhavan O, Ghaderi E, Aghayee S, Fereydooni Y, Talebi A (2012). The use of a glucose-reduced graphene oxide suspension for photothermal cancer therapy. J Mater Chem.

[36] Sheng Z, Song L, Zheng J, Hu D, He M, Zheng M, Gao G, Gong P, Zhang P, Ma Y, Cai L (2013). Protein-assisted fabrication of nano-reduced graphene oxide for combined in vivo photoacoustic imaging and photothermal therapy. Biomaterials.

[37] Capdevielle P, Lavigne A, Sparfel D, Baranne-Lafont J, Cuong NK, Maumy M (1990). Mechanism of primary aliphatic amines oxidation to nitriles by the cuprouschloride-dioxygen-pyridine system. Tetrahedron Lett.

[38] Lai L, Chen L, Zhan D, Sun L, Liu J, Lim SH, Pohb CK, Shena Z, Lin J (2011). One-step synthesis of NH_2_-graphene from in situ graphene-oxide reduction and its improved electrochemical properties. Carbon.

[39] Zhou Y, Bao QL, Tang LAL, Zhong YL, Loh KP (2009). Hydrothermal dehydration for the “green” reduction of exfoliated graphene oxide to graphene and demonstration of tunable optical limiting properties. Chem Mater.

[40] Lee JH, Park G, Hong GH, Choi J, Choi HS (2012). Design considerations for targeted optical contrast agents. Quant Imaging Med Surg.

[41] Zha Z, Deng Z, Li Y, Li C, Wang J, Wang S, Qu E, Dai Z (2013). Biocompatible polypyrrole nanoparticles as a novel organic photoacoustic contrast agent for deep tissue imaging. Nanoscale.

[42] Kulkarni SA, Feng SS (2013). Effects of particle size and surface modification on cellular uptake and biodistribution of polymeric nanoparticles for drug delivery. Pharm Res.

