# Supplementary material for: Indocyanine Green Loaded Reduced Graphene Oxide for In Vivo Photoacoustic/Fluorescence Dual-Modality Tumor Imaging
Source: Nanoscale Res Lett. 2016 Feb 12;11:85. doi: 10.1186/s11671-016-1288-x (PMC4751099; doi:10.1186/s11671-016-1288-x)
Supplement: Additional file 1: — The supplementary characterization results of rNGO-PEG/ICG. (DOCX 2405 kb) [file 11671_2016_1288_MOESM1_ESM.docx]

**Supplementary Material**

**Indocyanine Green Loaded** **Reduced Graphene Oxide for *in vivo* Photoacoustic/Fluorescence Dual-Modality Tumor Imaging**

Jingqin Chen^1†^, Chengbo Liu ^1^^2*†^, Guang Zeng ^1^, Yujia You ^3^, Huina Wang ^1^, Xiaojing Gong ^1^, Rongqin Zheng ^3^, Jeesu Kim^4^, Chulhong Kim ^4^, and Liang Song ^12*^

^1^ Research Laboratory for Biomedical Optics and Molecular Imaging, Shenzhen Key Laboratory for Molecular Imaging, Institute of Biomedical and Health Engineering, Shenzhen Institutes of Advanced Technology, Chinese Academy of Sciences, Shenzhen 518055, China.

^2^ Beijing Center for Mathematics and Information Interdisciplinary Sciences (BCMIIS), Beijing 100048, China

^3^ Department of Medical Ultrasound, The Third Affiliated Hospital of Sun Yat-sen University, Guangzhou 510630, China.

^4^ Departments of Creative IT Engineering and Electrical Engineering, Future IT Innovation Laboratory, Pohang University of Science and Technology (POSTECH), 77 Cheongam‑Ro, Nam‑Gu, Pohang, Gyeongbuk 790‑784, Republic of Korea

^†^ These two authors contributed equally.

*Correspondence: liang. song@siat.ac.cn; cb.liu@siat.ac.cn


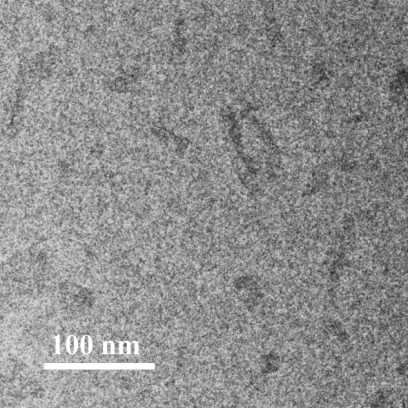


**A**


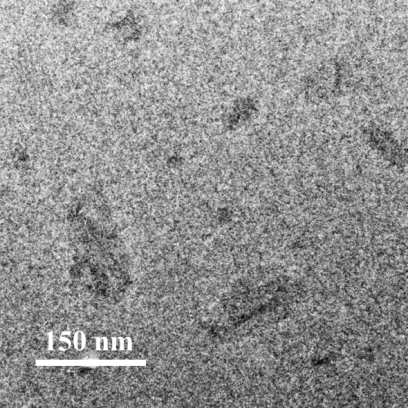


**B**

***Figure S1*** *The TEM images of rNGO-PEG/ICG before (A) and after (B) exposing under the 780 nm laser (6 mJ cm^−2^) for 30 min.*


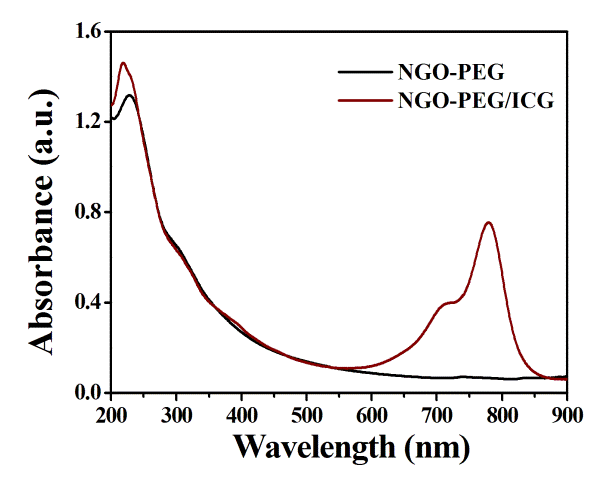


**ICG loading ratio (w/w %)**

**NGO-PEG rNGO-PEG**

**Figure S2**. (A) UV−vis absorption spectra of NGO-PEG, NGO-PEG/ICG solutions. (B) Comparison of the ICG loading capacity of NGO-PEG and rNGO-PEG. ***p*<0.01.


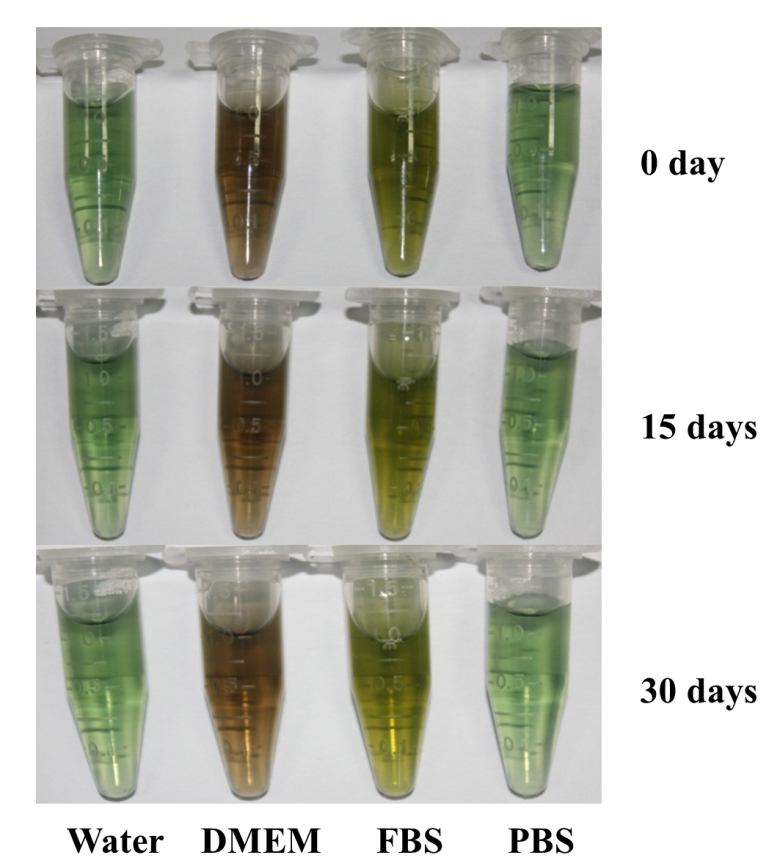


**Figure S3**. (A) Photographs of rNGO-PEG/ICG solution in different solvents after storage at 4 ^o^C for 1 month.

***

***

***Figure S4*** *The cell uptake ratio of free ICG and rNGO-PEG/ICG detected using the Flow cytometry.*
